# LED-based characterization of solar cells for underwater applications

**DOI:** 10.1016/j.xpro.2023.102833

**Published:** 2024-01-26

**Authors:** Alice Zhang, B. Edward Sartor, Jason A. Röhr, André D. Taylor

**Affiliations:** 1 Department of Chemical and Biomolecular Engineering, Tandon School of Engineering, New York University, Brooklyn, NY 11201, USA; 2 Singh Center for Nanotechnology, School of Engineering and Applied Sciences, University of Pennsylvania, Philadelphia, PA 19104, USA; 3 General Engineering, Tandon School of Engineering, New York University, Brooklyn, NY 11201, USA

**Keywords:** Physics, Energy

## Abstract

Improved solar energy harvesting in aquatic environments would allow for superior environmental monitoring. However, developing underwater solar cells is challenging as evaluation typically requires deployment in the field or in large water tanks that can simulate aquatic light conditions. Here, we present a protocol to test underwater solar cells using a light-emitting diode (LED)-based characterization technique usable in a typical laboratory setting. We describe steps for installing and running Python code, matching LEDs to irradiance, characterizing underwater solar cells, and calculating underwater solar cell efficiency.

For complete details on the use and execution of this protocol, please refer to Röhr et al.^1^

## Highlights

• Protocol for characterizing underwater solar cells in a laboratory setting
• Underwater solar irradiance is replicated using an LED solar simulator
• Improved characterization accuracy using a spectral mismatch factor

## Abstract (editor highlights)

Publisher’s note: Undertaking any experimental protocol requires adherence to local institutional guidelines for laboratory safety and ethics.

## Graphical abstract

**Figure:**
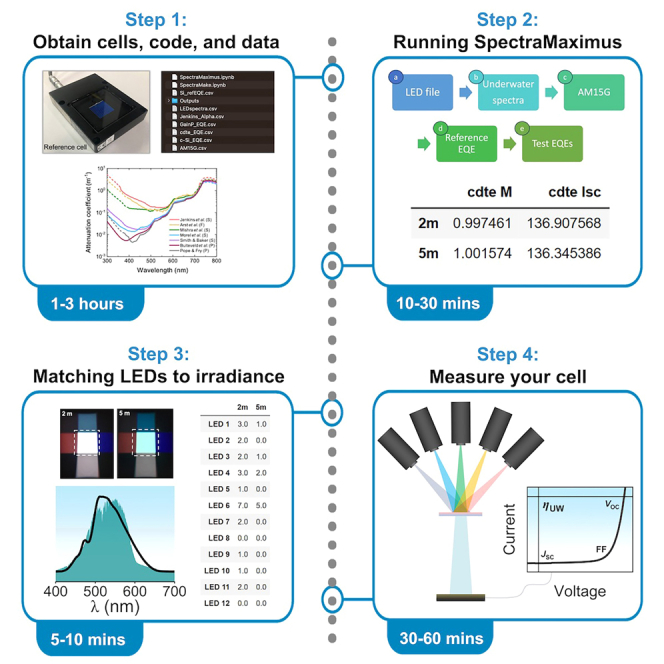

## Before you begin

This protocol describes how the power conversion efficiency (η) of an underwater solar cell can be characterized in a typical laboratory setting by simulating the underwater spectrum with an LED solar simulator. This type of solar simulator works particularly well for this purpose as it allows for tuning of the irradiance spectrum by varying the intensities of each individual LED (centered at different wavelengths). Using this feature, LEDs can be used to simulate underwater spectra and the performance of a solar cell can be measured under such conditions.

**Figure 1:**
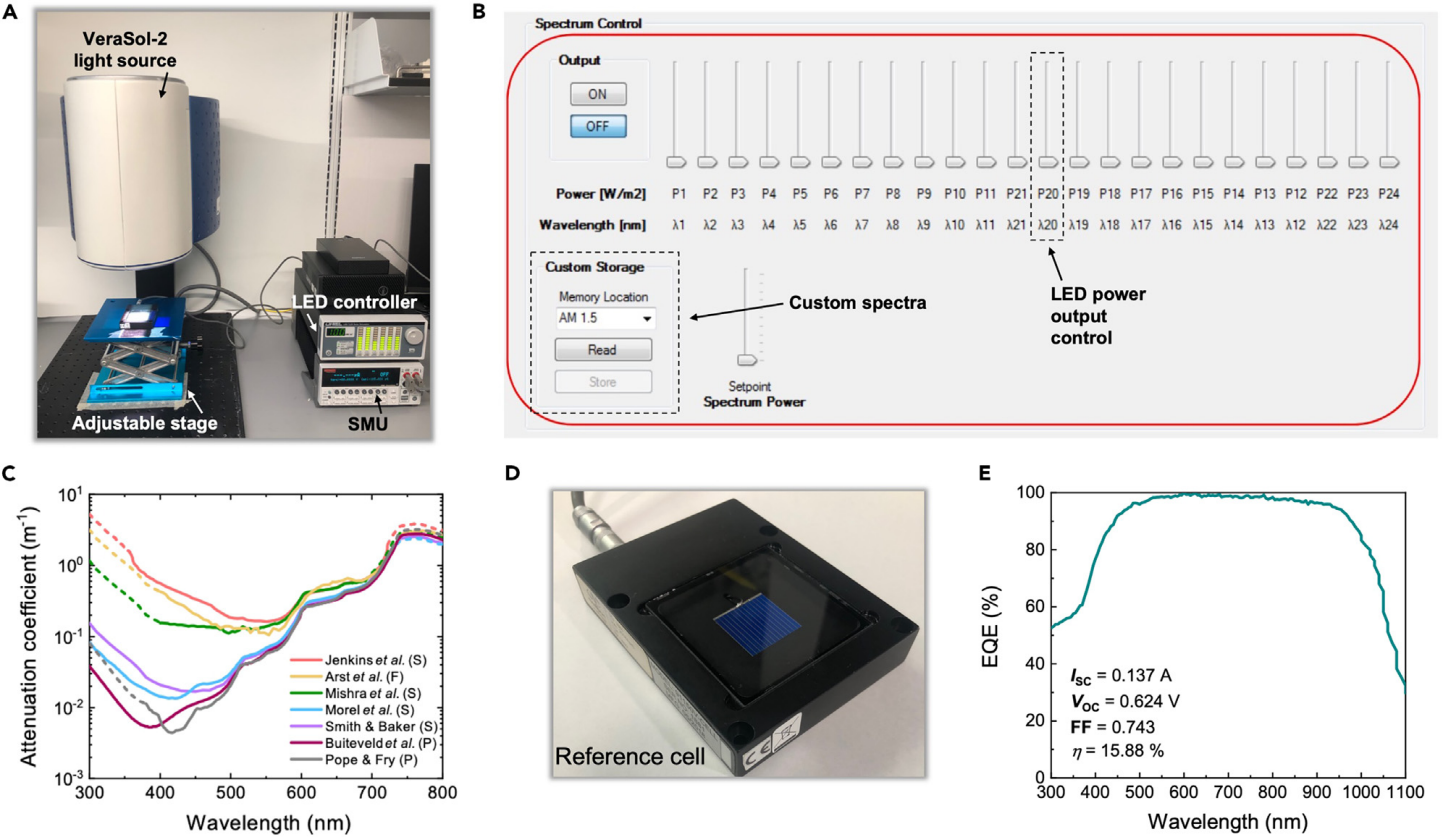
Before you begin (A) Photograph of the employed experimental setup consisting of the VeraSol-2 setup class AAA solar simulator coupled to a Keithley 2400 SMU. (B) Spectrum control software, allowing for fine tuning each individual LED in the setup. (C) Water-attenuation spectra of various saltwater (S), freshwater (F) and pure water (P) sources. (D) Newport Oriel mc-Si reference solar cell. (E) EQE spectrum and device characteristics of the mc-Si solar cell. (A, B) was adapted from Röhr et al.^1^; (C) was adapted from Röhr et al.^2^

While we employed an Oriel VeraSol-2 by Newport (Figure 1A), any LED solar simulator with LEDs in the spectral range of interest can, in principle, be employed for this purpose. The VeraSol-2 arrives with software allowing for easy adjustment of the output irradiance of each LED in the array (Figure 1B), which is important for simulating underwater irradiance spectra (Figure 1C). Additionally, up to nine custom irradiance spectra can be saved within the accompanying software, which reduces human error and allows for easy access between the underwater solar spectra. Besides the solar simulator, a source-measure unit (here a Keithley 2400 SMU), a laboratory PC, and a stage (here a lab jack) with an adjustable height are needed.

This protocol includes Python code used for calculating the power settings to match simulator LEDs to the underwater spectra, translation to reference conditions, and spectral mismatch calculations. Additional code for calculating the water-attenuated irradiance spectra has also been given if existing data is not available.

While all the used data can be found on the GitHub repository, below we provide steps as to how the data can be obtained directly from the primary sources. So, while not strictly necessary, we strongly encourage the reader to go through the steps outlined below.

### Acquire the reference and test solar cell’s EQE and device characteristics under terrestrial conditions

**Timing: 1–2 h (not including fabrication)**

This step allows for required inputs for the code SpectraMaximus to be obtained. There must be two solar cells available to calibrate the LED solar simulator, namely the test cell and the reference cell. The test cell, i.e., the solar cell that will be evaluated for underwater applications, will need to be fabricated or acquired. This cell’s characteristics under terrestrial AM1.5G conditions (especially its short circuit current, *I*_SC_) and its external quantum efficiency (EQE) spectrum must be measured if it is not already known. The same is true for the reference cell.

***Note:*** As this protocol only pertains to how to characterize underwater solar cells, instructions for how to fabricate a solar cell will not be provided.

1. Obtain the EQE and J-V curve of the reference cell under AM1.5G illumination.
  a. The reference cell we utilized can be bought directly on Newport’s website.
  b. Measure the EQE spectrum in a range of at least 300–1100 nm if not already known.
    ***Note:*** When measuring the reference cell, make sure that the EQE spectral shape matches that of the certified spectrum across the entire wavelength range. A bias light may be used to measure the EQE but is not required.
  c. Measure the reference cell’s characteristics under standard AM1.5G illumination, by performing a *J*-*V* scan, using the following well-known equation.
    (Equation 1) $$\eta =\frac{I_{\text{SC}}V_{\text{OC}}\text{FF}}{P_{\text{in}}}\times 100\;\%$$
    where $P_{\text{in}}$ is the total power of the AM1.5G spectrum. Record the reference cell device characteristics (most importantly the *I*_SC_) under these conditions if not already known.
  d. Ensure that all cell characteristics match the certified values.
    **CRITICAL:** While we do not recommend this, if a home-made reference cell is utilized, the above step is critically important to ensure reproducibility. The reference cell will be used to ensure accurate determination of the test cell characteristics. In our case, we used an encapsulated Newport Oriel micro-crystalline silicon (mc-Si) reference solar cell (Figure 1D) with well-known device characteristics and EQE spectrum (Figure 1E).
2. Measure the EQE of the test cell.
  a. Acquire the test cell to be measured under the simulated underwater irradiance.
    ***Note:*** For the sake of this protocol, this can be any test cell. While the reference cell can be utilized for this as well, the spectral mismatch calculations will be more interesting if a different type of solar cell is measured.
  b. Measure and record the EQE spectrum in a range of at least 300–1100 nm.
    ***Note:*** The VeraSol-2 might not fit inside a glovebox, so care should be taken if air-sensitive solar cells are measured. Encapsulation might be required. (Troubleshooting 1)

### Install python and download packages for *SpectraMaximus*

**Timing: 5–10 min**

All accompanying code provided by this protocol was written in Python. Therefore, Python will need to be installed and several packages must be downloaded. These packages will be used for any calculations related to this protocol. While any code editor or integrated development environment (IDE) can be used, Jupyter Notebooks (Anaconda) is highly recommended. Using the same IDE the code was developed in will help avoid issues with running the code.

3. Download *SpectraMaximus* from the GitHub repository.
4. Download the Anaconda distribution platform.
  a. Open Anaconda Navigator
  b. Launch *Jupyter Notebook.*
5. Locate the path of the downloaded protocol code (for example: C:∖Users∖User∖Downloads) and open the SpectraMaximus.ipynb file.
6. If Jupyter Notebook is being used for the first time, the *Python package index* (*pip*), which is used to install and manage software packages, will need to be upgraded to the latest version.
  a. Run the following:
    >!pip install notebook
    ***Note:*** Once pip is upgraded, pip will be used to install the following dependency packages for the code. These packages only need to be downloaded once and these lines of code can subsequently be commented out using # or deleted entirely.
  b. Run the following:

!pip install numpy

!pip install matplotlib

!pip install scipy

!pip install pandastable

!pip install sympy

!pip install math

### Obtaining AM1.5G irradiance and underwater-attenuated spectra

**Timing: 10 min to 1 h**

This step is used to obtain two spectra that is used within the code, namely the irradiance spectrum of the sun and the attenuation spectrum of the waters the solar cells are being evaluated for. Existing underwater-attenuated spectra ($\varepsilon_{UW})$ can be used or, if not already available, can be calculated by using the *SpectraMake* code.

7. Obtain $\varepsilon_{\text{UW}}$, either through existing data or calculating it by using the code *SpectraMake.* Here we describe how to calculate $\varepsilon_{\text{UW}}$ using *SpectraMake* if existing data is not available starting with obtaining water attenuation spectra.
  a. Download the latest version of the plot digitizer that is compatible with your computer’s OS (any plot digitizer software will do).
  b. Choose a spectrum to investigate such as Jenkins’ water attenuation spectrum.
    ***Note:*** Water attenuation spectra vary depending on geographical location and Jenkin’s water attenuation spectrum is not the only spectrum that can be used, as many others exist from different waters across the globe, such as the spectra provided by Morel et al. (South Pacific) and Mishra et al. (off the coast of Roatan island in Honduras). In the GitHub repository, all extended (250–3000 nm) version of Jenkins, Morel, and Mishra’s water attenuation spectra can be found, AM1.5G data, and *SpectraMake* can be found in the repository. In this protocol, we will employ the wavelength-dependent attenuation spectrum of clear sea water obtained off the coast of Key West, Florida, USA.^3^ Remember to cite the article by Jenkins et al.,^3^ Mishra et al.,^4^ or Morel et al.^5^ every time their data is used.
  c. Open the figure in the plot digitizer software and digitize the data. Other water attenuation spectra from different geographical locations can be obtained in the same manner.
  d. The extension method and more examples of water attenuation spectra is described in Röhr et al.^2^
8. Obtain the AM1.5G solar irradiance spectrum $\left(\varepsilon_{\text{AM}1.5G}\right)$ from a source such as NREL.
  a. Under “Data Files”, click the “spreadsheet” or “compressed format” links to obtain access to the datafile.
  b. Read and agree to the “NREL data disclaimer”.
  c. Open the datafile and copy the (Wavelength [nm]) and Global tilt [W m^−2^ nm^−1^]) data columns.
9. Download *SpectraMake* from the GitHub repository.
10. Open *SpectraMake* in Jupyter Notebook.

***Note:*** In *SpectraMake*, the Beer-Lambert law, is used in conjunction with the water attenuation coefficient to calculate what the solar irradiance will look like at a specific underwater depth, where *α*(*λ*) is the wavelength-dependent attenuation spectrum of water and *D* is the water depth below sea level. The AM1.5G spectrum will be referred to as “0m” as it is on the surface of the water, or sea level.

(Equation 2) $$\varepsilon_{\text{UW}}\left(\lambda ,D\right)=\varepsilon_{\text{AM}1.5G}\left(\lambda \right)e^{-\alpha \left(\lambda \right)D}$$

11. Run *SpectraMake.*

dfam15g = fd.askopenfilename()

dfalpha = fd.askopenfilename()

xnew = np.linspace(300,1100, num=801) #range of wavelegth

depth_range = 9

**Figure 2:**
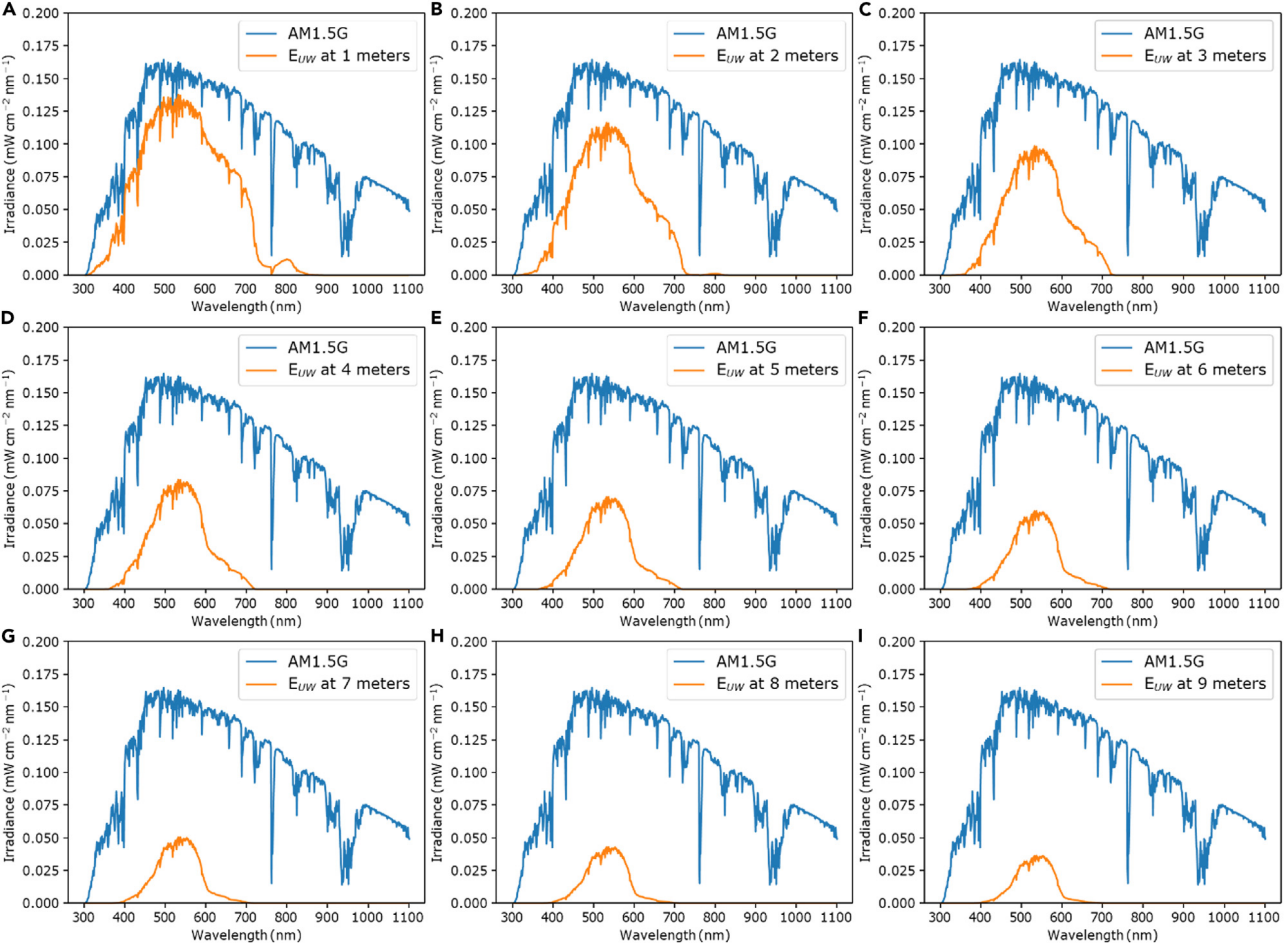
(A-I) *SpectraMake* output for creating $\varepsilon_{UW}$ Correction - AM1.5G irradiance spectrum (blue) and, $\varepsilon_{UW}\text{,}$ (orange) at 1–9 m below sea level

***Note:*** The required inputs for this code will be the data files for AM1.5G and water attenuation spectra. The files provided in the repository are from 250–3000 nm, however if other files are used, be sure to double check the wavelength range. In this protocol, a range of 300–1100 nm will be used. depth_range is the range from 1 to depth_range. For example, if 9 is used as an input, $\varepsilon_{\text{UW}}$ graphs will be generated for 1–9 meters underwater (Figures 2A–2I).

## Key resources table

**Table:** 

REAGENT or RESOURCE	SOURCE	IDENTIFIER
**Other**		

LED solar simulator	Newport/Oriel Instruments	VeraSol-2, class AAA
Source measure unit	Keithley	2400S-900-01K
mc-Si reference solar cell	Newport/Oriel Instruments	Oriel 91150V
Quantum efficiency measurement kit	Newport/Oriel Instruments	QEPVSI-b
Laboratory PC	N/A	N/A
Adjustable stage/lab jack	N/A	N/A
Cables	N/A	N/A

**Software and algorithms**		

**Jupyter Notebook**	Anaconda	**N/A**
SpectraMaximus	This article	https://github.com/EdSartor/Solar
SpectraMake (optional)	This article	https://github.com/EdSartor/Solar

## Step-by-step method details

### Using SpectraMaximus

**Timing: 10–30 min**

Herein we describe step-by-step methods from loading raw data, matching the $\varepsilon_{\text{UW}}$ with LED solar simulators, determining spectral mismatch (M), and determining the short-circuit current correction ($I_{\text{SC},\text{LED}}^{\text{RC},M})$. This user-friendly code will only need a few inputs to be able to run. The following are the input files and reference cell $I_{\text{SC}}\;\left(I_{\text{SC}}^{\text{RC}}\right)$, here 136.56 mA, required to run this code. The underlying math used for the spectral-mismatch corrections is summarized at the end of this paper.

LED_file = fd.askopenfilename()

UnderwaterSpectra = fd.askopenfilenames()

AM15g = fd.askopenfilenames()

Reference_EQE = fd.askopenfilename()

Test_EQEs = fd.askopenfilenames()

Isc_Reference = 136.56

1. Download the *SpectraMaximus* code from the GitHub repository.
2. Run the *SpectraMaximus* code.

**Figure 3:**
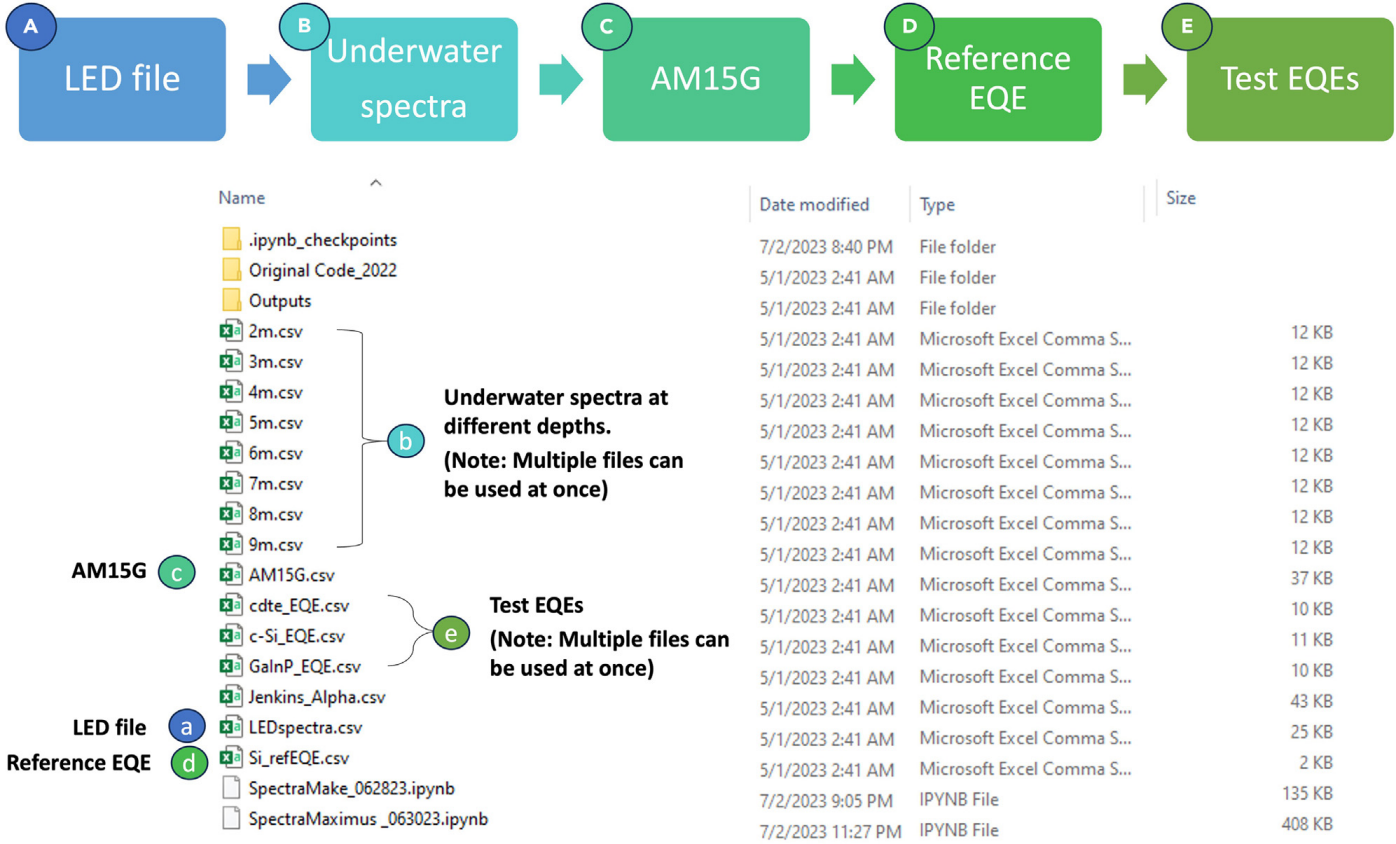
Step by step method for inputting files into SpectraMaximus.

***Note:*** This section of the code will open a separate window asking for file inputs for each variable. After the file(s) are chosen for one variable, subsequent windows will open until each of the five variables have a file input selected to be associated with it. Multiple tests can be run at the same time and more than one file can be inputted for LED_file, Spectra, and Test_EQEs. To illustrate this, we use CdTe and c-Si data to determine the corrected short circuit at 2- and 5-meters depth using the files provided in the GitHub repository. Once this section of code is running the following window will open and the order of inputs is shown below (Figure 3).

3. Once the desired input is chosen, click Open and proceed to the next file(s).
  a. Once this section of code is running, the first input will be the LED file, here LEDspecta.csv.
  b. The next will be the underwater spectrum or spectra that will be studied.
  c. Next will be the AM1.5G file.
  d. Subsequently, the reference cell EQE spectrum will be chosen.
  e. Finally, the test cell(s) EQE spectrum/spectra will be chosen.

***Note:*** All graphs, calculations, and data files are completed at the same time. This code might take a couple minutes to run if there are multiple files chosen at the same time.

**Figure 4:**
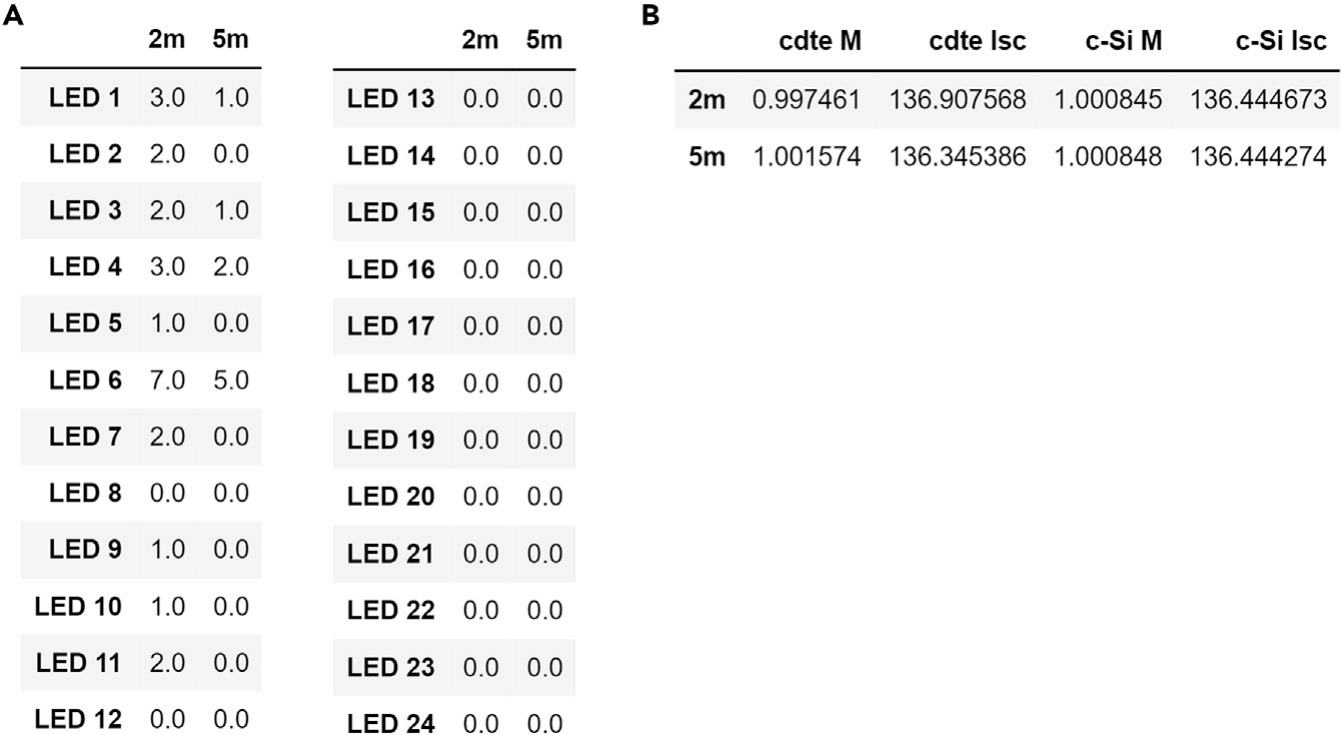
*SpectraMaximus* Outputs (A) LED Outputs (B) Spectral Mismatch and Current Corrected-*I*_SC_

4. An output of the calculation results is given in Figure 4, which shows the spectral mismatch coefficient, the mismatch-corrected reference solar cell translation value, and the total power density of the LED irradiance spectrum.

***Note:****M* was calculated to 0.997 and 1.002, respectively, for CdTe indicating that the simulated irradiance spectrum is comparable to the underwater irradiance spectrum, and that only a small value is needed to account for the differences in spectral response between the two cells. The same can be said of the c-Si device.

5. Accounting for the spectral mismatch, the height of the lab jack can now be adjusted such that the reference cell (which had *I*_SC_ of 136.56 mA under AM1.5G irradiance) yields an *I*_SC_ of 136.91 mA for the CdTe devices under LED irradiance simulating the underwater light at 2 m and 136.34 mA at 5 m.

### Matching LEDs to the underwater spectrum

**Timing: 5–10 min**

Some simulators, such as the G2V Pico LED Solar Simulator, allow for the direct replication of a spectrum from a file. However, other simulators, such as the VeraSol-2 Class AAA Solar Simulator used in this study, require manual calculation and setting of the LEDs for replication of a given spectrum. This step calculates LED input settings for the VeraSol-2 LED solar simulators to replicate a given spectrum.

**Figure 5:**
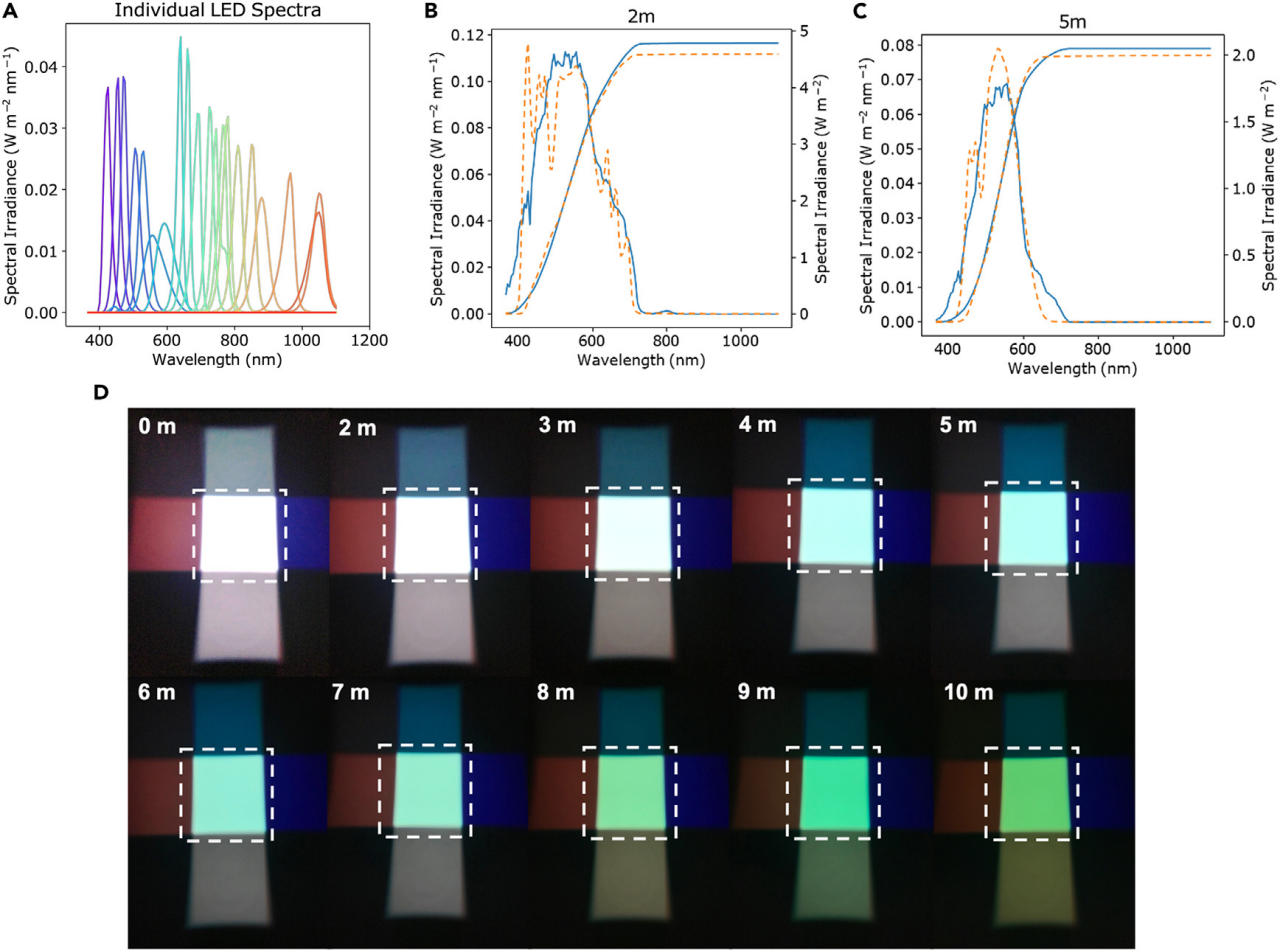
Underwater spectrum matching with LEDs (A) Baseline outputs for individual LEDS. (B and C) Target spectrum (orange) and output spectrum based on calculated values from *SpectraMaximus* python script (blue). The dashed lines are integrated Watts per meter^3^ and are used to define the quality of the fit. (D) Photograph of resulting simulated underwater LED light; adapted from Röhr et al.^1^

6. Download “LEDspectra.dat” from the repository (Figure 5A).

**CRITICAL:** If using an alternative LED Solar Simulator to the VeraSol-2 Class AAA Solar Simulator, an alternative LED file that contains in the first column wavelength, and in subsequent columns the irradiance per wavelength for each individual LED in the simulator will need to be determined and inputted instead.

7. Download desired output $\varepsilon_{\text{UW}}$.
  a. Download previously calculated $\varepsilon_{\text{UW}}$ from repository folder “Jenkins”. These spectra were calculated using the methods described in how to use *SpectraMake* and are labeled by depths (2m.csv, 3m.csv, etc.)
8. The output of this portion of the code is $\varepsilon_{\text{LED}}$, the lab-setting test spectrum. Later this will be used in the spectral mismatch code to account for any discrepancies (Figures 5B and 5C) seen between $\varepsilon_{\text{LED}}$ and $\varepsilon_{\text{UW}}$. The color of the light output is shown in Figure 5D for depths ranging from 0 to 10 m.
9. Set the LED powers to the numbers provided in the files labeled 2m.csv, etc.

### LED-based solar cell characterization

**Timing: 30 min to 1 h (not including fabrication)**

With all calculations done, you can now perform your solar cell characterization, and calculate the resulting solar cell efficiency, η.

**Figure 6:**
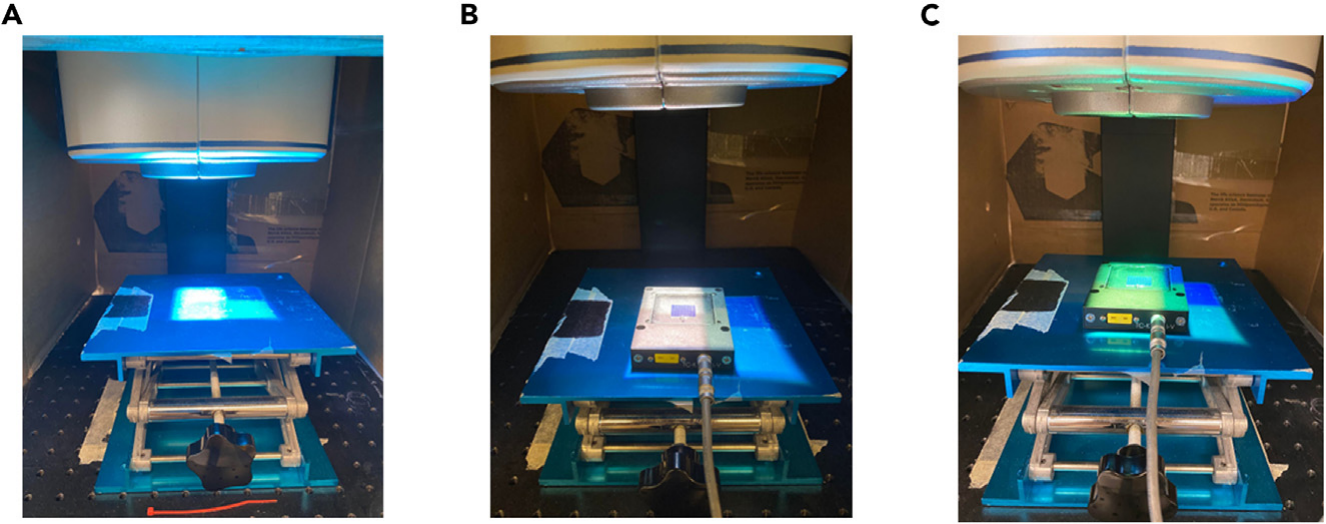
LED-based solar cell characterization set-up (A) Photograph of the solar simulator turned on and set to AM1.5G with no height adjustments made. (B) The height of the lab jack adjusted such that the *I*_SC_ of the mc-Si reference cell is measured at 136.56 mA (C), The mc-Si reference cell under simulated conditions for 5 m underwater light. The lab jack was adjusted to ensure that the *I*_SC_ was consistent with the calculated $I_{\text{REF}}^{\text{LED}}$ value.

10. Turn on the VeraSol-2 LED solar simulator and let the system warm up for ∼15 min (Figure 6A).

**CRITICAL:** If the solar simulator is covered with light-blocking fabric, make sure the cooling vents are not impeded as this might damage the LEDs.

11. Place the reference solar cell beneath the light source on the lab jack (Figure 6B).

**CRITICAL:** As the illuminated area from the VeraSol-2 is relatively narrow, it is important to ensure that the reference (and test cell for that matter) is placed in the same location between each measurement.

12. Set the LED spectrum to the one corresponding to AM1.5G and measure the *I*_SC_ of the reference cell (Figure 6B). Lower or raise the lab jack until the *I*_SC_ matches the verified value of the reference cell. Do not vary the light intensity of the lamp during this step.
  a. Take note of the stage height that gives the correct *I*_SC_ for AM1.5G illumination. This height should not vary significantly from day to day. If it does, then this could be a sign of LED degradation.

***Note:*** To ensure reproducibility, the same reference cell will need to be used, allowing data to be acquired in a consistent manner. A reference cell that has already been measured and verified is recommended for the most accurate acquisition of data.

13. Once the verified *I*_SC_ is reached, switch the AM1.5G spectrum to the underwater irradiance spectrum corresponding to the desired depth within the VeraSol-2 software (Figure 6C).

***Note:*** The LEDs will need to be adjusted manually or loaded from an existing spectrum on the VeraSol2 simulator software.

14. Adjust the lab jack to the height that yields the calculated *I*_SC_ value for the reference cell, accounting for spectral mismatch (Figure 4).
15. Once the lab jack is in the correct position, replace the reference cell with the test cell and perform a *J*-*V* scan while illuminating the solar cell. (Troubleshooting 3).

**CRITICAL:** Cover the entire solar simulator with light-blocking fabric or place the solar simulator in a dedicated dark room. The underwater irradiance spectra have very low intensity, so covering the simulator will help ensure that no outside light can interfere with the measurements. (Troubleshooting 2)

16. The solar cell characteristics can now be calculated using Equation 1.

***Note:*** For traditional solar cell measurements, $p_{\text{LED}}$ is replaced by the total power density of the AM1.5G spectrum. Since $p_{\text{LED}}$ is always lower than that for the AM1.5G, a higher efficiency can typically be observed for underwater solar cells than what would be expected for terrestrial solar cells.^1^^,^^2^

## Expected outcomes

This protocol provides the python code and experimental procedure for measuring underwater solar cells using an LED solar simulator. The provided code is used to both calibrate the LEDs to accurately simulate underwater light conditions and translate the reference solar cell to the proper reference conditions, accounting for spectral mismatch.

We show a particular example where we want to characterize a CdTe solar cell at 5 m depth below sea level (here simulating water off the coast of Key West, Florida, US), using a calibrated mc-Si solar cell as a reference cell. We give a detailed explanation of the calculations needed to calibrate the experimental setup and show how the solar cell is subsequently characterized. In this particular example, we show what the LEDs should be set to and what the *I*_SC_ of the reference cell should be set to in order to ensure proper reference conditions (Figure 6).

## Quantification and statistical analysis

### Math used for the current-correction procedure

The simulated spectrum deviates slightly from the desired underwater spectrum, requiring a calibration step to accurately simulate the current characteristics of the test device. This calibration is easily accomplished by adjusting the distance between the device and the simulator using an adjustable lab jack and the calibrated reference device.

The provided code, *SpectraMaximus,* takes the LED outputs, underwater spectra, reference spectrum (AM1.5G), reference device EQE, and test device EQE to calculate a target short circuit current for the reference cell, at which height the following relation is true:

(Equation 3) $$\frac{I_{\text{DUT}}^{\text{LED}}}{I_{\text{DUT}}^{\text{UW}}}=1$$

By defining a spectrum calibration number, *CN*, for the underwater and reference spectra as in Osterwald et al.^6^ we define the current of the reference cell under an arbitrary underwater spectrum. Dividing these two equations together obtains Equation 5.

(Equation 4) $${CN}^{UW}=\frac{I_{REF}^{UW}}{E_{UW}},{CN}^{RC}=\frac{I_{REF}^{RC}}{E_{tot}^{x}}$$

(Equation 5) $$I_{REF}^{UW}=I_{REF}^{RC}\;\frac{{CN}^{UW}\times E_{UW}}{{CN}^{RC}\times E_{tot}}$$

Substituting integrated spectral response and integrated spectral irradiance into the equations for calibration number, we obtain:

(Equation 6) $${CN}^{RC}=\frac{\int_{\lambda_{1}}^{\lambda_{2}}SR_{\text{REF}}\left(\lambda \right)\times \varepsilon_{\text{REF}}\left(\lambda \right)\;d\lambda }{\int_{\lambda_{1}}^{\lambda_{1}}\varepsilon_{\text{RC}}\left(\lambda \right)\;d\lambda }$$

(Equation 7) $${CN}^{UW}=\frac{\int_{\lambda_{1}}^{\lambda_{2}}SR_{\text{REF}}\left(\lambda \right)\times \varepsilon_{\text{UW}}\left(\lambda \right)\;d\lambda }{\int_{\lambda_{1}}^{\lambda_{1}}\varepsilon_{\text{UW}}\left(\lambda \right)\;d\lambda }$$

By substituting these equations into Equation 5 and canceling the appropriate terms, we obtain a simple ratio of two integrals multiplied by the reference current to obtain $I_{REF}^{UW}\;\text{.}$

(Equation 8) $$I_{\text{REF}}^{\text{UW}}=I_{\text{REF}}^{\text{RC}}\times \frac{\int_{\lambda_{1}}^{\lambda_{2}}SR_{\text{REF}}\left(\lambda \right)\times \varepsilon_{\text{UW}}\left(\lambda \right)\;d\lambda \;}{\int_{\lambda_{1}}^{\lambda_{2}}SR_{\text{REF}}\left(\lambda \right)\times \varepsilon_{\text{RC}}\left(\lambda \right)\;d\lambda }$$

Having obtained the relevant underwater current for the reference device, we must now calculate the spectral mismatch^7^ coefficient *M* between the underwater spectrum and the simulated LED spectrum. *M* is defined as the ratio of four currents in Osterwald et al.^6^ We solve for the desired current $I_{REF}^{\text{LED}}$ and apply the current matching condition in Equation 12.

(Equation 9) $$M=\frac{I_{\text{DUT}}^{\text{LED}}}{I_{\text{REF}}^{\text{LED}}}\times \frac{I_{\text{REF}}^{\text{UW}}}{I_{\text{DUT}}^{\text{UW}}}$$

(Equation 10) $$M=\frac{\int_{\lambda_{1}}^{\lambda_{2}}\varepsilon_{\text{LED}}\left(\lambda \right)\times {SR}_{\text{DUT}}\left(\lambda \right)\;d\lambda }{\int_{\lambda_{1}}^{\lambda_{2}}\varepsilon_{\text{LED}}\left(\lambda \right)\times {SR}_{\text{REF}}\left(\lambda \right)\;d\lambda }\times \frac{\int_{\lambda_{1}}^{\lambda_{2}}\varepsilon_{\text{UW}}\left(\lambda \right)\times {SR}_{\text{REF}}\left(\lambda \right)\;d\lambda }{\int_{\lambda_{1}}^{\lambda_{2}}\varepsilon_{\text{UW}}\left(\lambda \right)\times {SR}_{\text{DUT}}\left(\lambda \right)\;d\lambda }\text{.}$$

**Figure 7:**
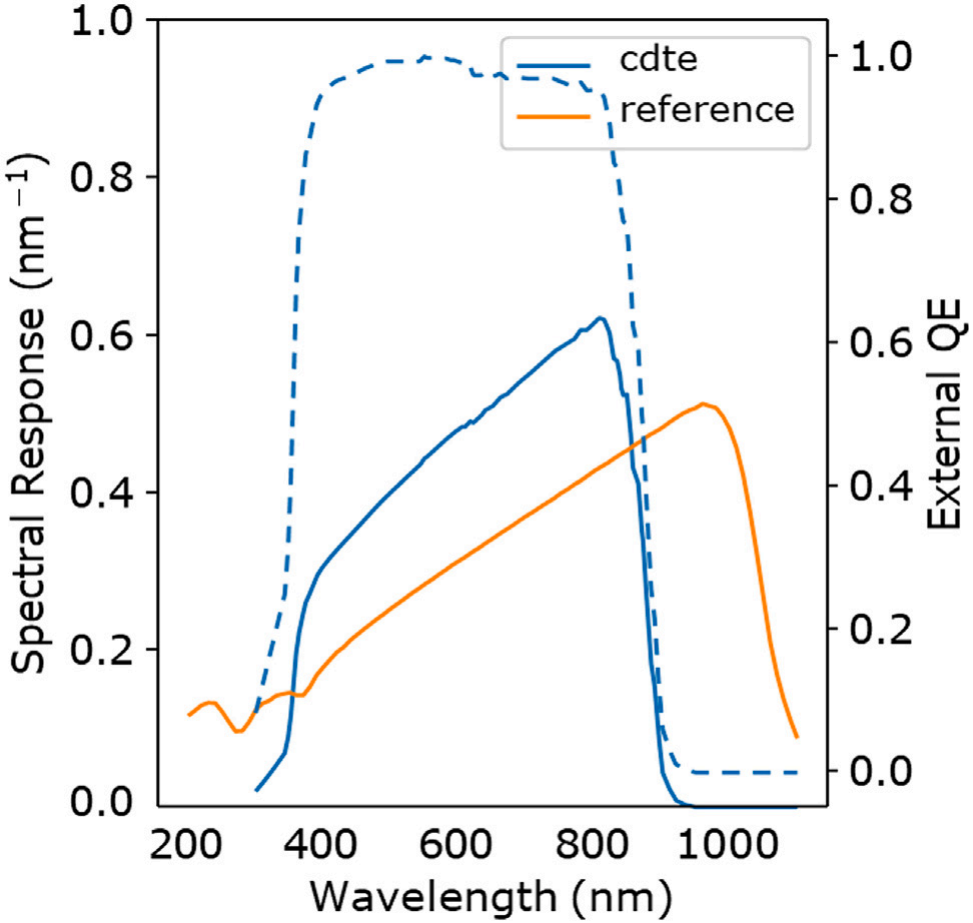
Spectral response and EQE ${SR}_{DUT}$ of the CdTe cell (blue) and the corresponding EQE (dotted) compared to ${SR}_{RC}$ of the mc-Si reference cell.

SR stands for spectral response and it is related to the cell’s EQE, which is one of the initial inputs into *SpectraMaximus*. The conversion of the reference and test cell EQEs to ${SR}_{REF}$ and ${SR}_{DUT}$ (Figure 7) is found by the following equation, where *q* is the elementary charge, λ is the wavelength, *h* is Planck’s constant, and *c* is the speed of light:

(Equation 11) $$SR\left(\lambda \right)=\frac{q\lambda }{hc}\text{EQE}\left(\lambda \right)=\frac{\lambda }{1239.8}\text{EQE}\left(\lambda \right)$$

for i in range (0,len(Test_EQEs)):

 test= pd.read_csv(Test_EQEs[i],header=0,names=['wv','qe'])

 if i == 0:

  df_EQE['wv'] = test['wv']

 test['sr'] = ((test['qe'])∗(test['wv'] / 1239.8))

When Equation 9 is manipulated to isolate for $I_{REF}^{\text{LED}}$, the obtained value is the short-circuit current that the reference device will register when it is at the height for which the current matching condition (Equation 3) is true, ensuring an accurate current is measured in the test device.

(Equation 12) $$I_{REF}^{\text{LED}}=\frac{I_{\text{REF}}^{\text{UW}}}{M}$$

The code describing the above math is shown below.

for Wavelength_name in name_list:

 Simulation_spectrum = np.dot(L,df_LEDset[wv_name])

 Wavelength_range = np.linspace(start_nm, end_nm,∖

 num=(end_nm-start_nm+1))

 #---------------Interpolation ----- ----------------

 # . . . Refer to code . . . |

 # --------------------------------------------------

 #----------Spectral Mismatch Calculation -----------

 . . .

 int1 = np.trapz(SR_ref ∗ E_uws)

 int2 = np.trapz(SR_ref ∗ E_sim)

 int3 = np.trapz(SR_test ∗ E_sim)

 int4 = np.trapz(SR_test ∗ E_uws)

 int5 = np.trapz(SR_ref ∗ E_am15g)

 h = int3/int4

 E_sim = E_sim/h

      . . .

#-------------Spectral Mismatch Calculation ---------

  m = (int1/int2) ∗ (int3/int4)

      . . .

#--------Short Circuit Current Correction------------

  I_uw_ref = I_rc_ref∗(int1/int5)

  I_sim_ref = I_uw_ref/m

## Limitations

We show that underwater light conditions similar to what is expected off the coast of Key West, Florida, US, can be simulated with reasonable accuracy using the LED solar simulator. To simulate water in other regions, the LEDs might need to be shifted within the spectral range and/or the number of LEDs in the solar simulator might need to be expanded to include more LEDs in the UV part of the spectrum. For real-world applications, variations in temperature along with turbulent water conditions will affect solar cell performance, neither of which was accounted for here. Temperature control could be included into the setup, by adding cooling elements to the sample stage; however, the effect of waves is less trivial to include. Waves and marine biological activity can be expected to sway an underwater solar device, giving rise to variations in the amount of light hitting the solar cell which can also change the spectrum of incident light on the solar cells. Creating a stand that could shift in angle, along with filters simulating diffused underwater light, could increase the accuracy of the presented method.

## Troubleshooting

### Problem 1

Variations in results may occur due to solar cell degradation. Some solar cells are air sensitive and cannot be exposed to oxygen or moisture. If the device is taken out of the glovebox, potential shifts in data could occur due to the degradation/oxidation of the material.

### Potential solutions

• Encapsulating the device with glass and a sealant in a glovebox could protect the device from outside factors.
• Perform the protocol within a glovebox.

### Problem 2

It is often fine to measure solar cells in a lit room as the intensity of the solar simulator (AM1.5G equivalent) will far outweigh the intensity of the ambient light. However, the measurements herein can be very sensitive to ambient light as they are often under much lower intensity than the ambient. If care is not taken, overexposure of your cell can lead to wrong estimates of underwater efficiency.

### Potential solutions

• Perform all measurements in a dedicated dark room. Ensure that computer monitors, and other equipment, are turned off during measurements so that no unwanted light reaches the cell.
• Place the measurement setup inside a dedicated box. Ensure that the setup is well-ventilated and that no unwanted light enters. Cracks can be covered with light-absorbing, non-reflective matt black tape.

### Problem 3

Alligator clips or electrical wires are often used in obtaining the J-V data. In some instances, the wrong connections can cause the J-V curve displayed on the screen to look noisy or jagged.

### Potential solutions

• Ensure that the ground (typically black) and “live” wire (typically red) is correctly placed and there is no damage to the wires.

### Problem 4

The code is working, but the calculation does not seem correct.

### Potential solutions

• Double check the units of the input files.
• Double check if the wavelength ranges are equivalent.
• Restart the kernel and run the code again.

## Resource availability

### Lead contact

Further information and requests for resources and reagents should be directed to and will be fulfilled by the lead contact, André D. Taylor (andre.taylor@nyu.edu).

### Technical contact

Technical questions about this protocol should be directed to the technical contact, Jason A.Röhr (jasonrohr@nyu.edu).

### Materials availability

This study did not generate new unique reagents.

### Data and code availability

1. Any data will be shared by the lead contact upon request.
2. All original code has been deposited at GitHub (https://github.com/EdSartor/Solar) and Zenodo (https://doi.org/10.5281/zenodo.10308332), and is publicly available as of the date of publication.
3. Any additional information required to reanalyze the data reported in this paper is available from the lead contact upon request.
